# Supplementary material for: Experimental proof of the reciprocal relation between spin Peltier and spin Seebeck effects in a bulk YIG/Pt bilayer
Source: Sci Rep. 2019 Feb 14;9:2047. doi: 10.1038/s41598-019-38687-4 (PMC6376020; doi:10.1038/s41598-019-38687-4)
Supplement: Supplementary file 1 — Supplementary information for Experimental proof of the reciprocal relation between spin Peltier and spin Seebeck effects in a bulk YIG/Pt bilayer [file 41598_2019_38687_MOESM1_ESM.pdf]

Supplementary information for

# *Experimental proof of the reciprocal relation between spin Peltier and spin Seebeck effects in a bulk YIG/Pt bilayer*

Alessandro Sola(1), Vittorio Basso(1), Michaela Kuepferling(1), Carsten Dubs(2), Massimo Pasquale(1)

*(1) Istituto Nazionale di Ricerca Metrologica, Strada delle Cacce 91, 10135, Torino, Italy*
*(2) INNOVENT e.V., Technologieentwicklung, Prüssingstrasse 27B, 07745 Jena, Germany*

August 29, 2018

## Supplementary note 1: Thermal equations for the spin Peltier effect

The equivalent thermal circuit of Fig.2 of the paper is the basis for the measurement analysis and evaluation of the spin Peltier effect (SPE). The derivation of the elements of the circuit is based on the theory presented in Refs.[1, 2]. Here we summarize the derivation of the equivalent circuit and we apply it to this specific experimental case.

In YIG the heat current is

$$I_{q,h} = -\frac{1}{\mathcal{R}_{YIG}}\left[(T_2 - T_0) - \Delta T_{SP}\right] \tag{1}$$

where

$$\mathcal{R}_{YIG} = \frac{1}{\kappa_{YIG}}\frac{t_{YIG}}{A_{YIG}} \tag{2}$$

is the thermal resistance of YIG, where $\kappa_{YIG}$ is the bulk thermal conductivity and $A_{YIG}$ is its cross sectional area and $\Delta T_{SP}$ is the spin Peltier temperature difference which is given by the expression

$$\Delta T_{SP} = -\theta_{SH}\mu_0\left(\frac{\mu_B}{e}\right)\frac{1}{v_p}\frac{\epsilon_{YIG}\sigma_{YIG}T}{\kappa_{YIG}}j_{e,y} \tag{3}$$

where $\theta_{SH}$ is the spin Hall angle of Pt, $\epsilon_{YIG}$ is the thermomagnetic power coefficient of YIG, $\sigma_{YIG}$ is the magnetic moment conductivity of YIG, $\mu_B$ is the Bohr magneton and $e$ is the elementary change. $v_p$ is the magnetic moment conductance per unit surface area of the YIG/Pt interface and depends on the intrinsic conductances, $v_M$ of YIG and of Pt, on the ratio $t/l_M$ between the thickness $t$ and the diffusion length $l_M$, for each layer. The expression of $v_p$ is derived in Ref.[2] and reported in the Supplementary note 2. $T$ is the absolute temperature (average value along the YIG) and $j_{e,y}$ is the electric current density in Pt.

The thermal problem in Pt, by neglecting, as a first approximation, the dissipation due to magnetic moment currents, corresponds to the equation

$$\nabla_x^2 T = -\frac{j_{e,y}^2}{\sigma_e\kappa_{Pt}} \tag{4}$$

where $\sigma_e$ is the electric conductivity of Pt and $\kappa_{Pt}$ is its thermal conductivity. With the boundary conditions as in Fig.2, the following heat currents at the interfaces are obtained

$$I_{q,h} = -\frac{1}{\mathcal{R}_{Pt}}(T_0 - T_1) + \frac{1}{2} I_{q,JH} \tag{5}$$

$$I_{q,c} = -\frac{1}{\mathcal{R}_{Pt}}(T_0 - T_1) - \frac{1}{2} I_{q,JH} \tag{6}$$

where

$$\mathcal{R}_{Pt} = \frac{1}{\kappa_{Pt}} \frac{t_{Pt}}{A_{Pt}} \tag{7}$$

is the thermal resistance of Pt and $A_{Pt}$ is its cross section area. The Joule heat is $I_{q,JH} = R_{Pt} I_e^2$ and $R_{Pt} = \sigma_e^{-1} L_{e,y}/(L_z t_{Pt})$ is the electrical resistance of the Pt layer.

The description of the thermal problem of the SPE experiment is completed by the addition of the thermal contact resistances $\mathcal{R}_{cont,c}$ and $\mathcal{R}_{cont,h}$ (which also include the thermal resistance of the heat sensors) with equations

$$T_c = T_1 + \mathcal{R}_{cont,c} I_{q,c} \tag{8}$$

$$T_h = T_2 - \mathcal{R}_{cont,h} I_{q,h} \tag{9}$$

The previous equations corresponds to the equivalent thermal circuit represented in Fig.2 of the paper.

In the specific case of the spin Peltier measurement under isothermal conditions, i.e. $T_h = T_c = T$, we have

$$I_{q,c} = \Delta T_{SP}/\mathcal{R} - (\mathcal{R}_h/\mathcal{R}) I_{q,JH} \tag{10}$$

$$I_{q,h} = \Delta T_{SP}/\mathcal{R} + (\mathcal{R}_c/\mathcal{R}) I_{q,JH} \tag{11}$$

where the total resistances at each side are $\mathcal{R}_h = \mathcal{R}_{Pt}/2 + \mathcal{R}_{YIG} + \mathcal{R}_{cont,h}$ and $\mathcal{R}_c = \mathcal{R}_{cont,c} + \mathcal{R}_{Pt}/2$ and $\mathcal{R} = \mathcal{R}_h + \mathcal{R}_c$.

Once the two heat currents, $I_{q,c}$ and $I_{q,h}$, are simultaneously measured, the Joule heat is directly given by the difference

$$I_{q,JH} = I_{q,h} - I_{q,c} \tag{12}$$

while the spin Peltier $\Delta T_{SP}$ directly results as

$$\Delta T_{SP} = \mathcal{R} I_{q,s} + \frac{\mathcal{R}_h - \mathcal{R}_c}{2} I_{q,JH} \tag{13}$$

where

$$I_{q,s} = \frac{I_{q,h} + I_{q,c}}{2} \tag{14}$$

is the half sum.

In principle the SPE can be determined by Eq.(13), however the direct use of the Eq.(13) can be problematic. Even if one would be technically able to achieve an $\mathcal{R}_c - \mathcal{R}_h$ difference very close to zero, the Joule heat contribution $I_{q,JH}$, that can be several orders of magnitude larger than the spin Peltier heat, will not permit to determine $\Delta T_{SP}$ with the required resolution. In order to accurately subtract the Joule heat we then exploit the odd parity of the SPE with respect to the inversion of $I_{e,y}$ along $y$ or of the magnetization along $z$. If the sign switching of the current or of the magnetic field is fast enough, the Joule heat offset remains stationary in time and can be automatically eliminated by the subtraction. Then taking $I_{q,s}$ as defined in Eq.(14), when the applied field is $H_s$ is inverted in sign, we compute

$$I_{q,SP} = \frac{I_{q,s}(H_s) - I_{q,s}(-H_s)}{2} \tag{15}$$

Similarly under the inversion of the sign of the electric current we compute

$$I_{q,SP} = \frac{I_{q,s}(I_{e,y}) - I_{q,s}(-I_{e,y})}{2} \tag{16}$$

In both cases the temperature difference can be directly computed as $\Delta T_{SP} = \mathcal{R} I_{q,SP}$ by the knowledge of the total thermal resistance $\mathcal{R}$.

# Supplementary note 2: Magnetic moment conductance at the YIG/Pt interface

The transmission of the magnetic moment current between the two layers is determined by the diffusion length $l_M$ and by the intrinsic conductance $v_M$ of each layer and by the thicknesses $t_{YIG}$ and $t_{Pt}$ of YIG and Pt. From Ref.[1, 2] the magnetic moment conductances for the bilayer is

$$\frac{1}{v_p} = \frac{v_{eff}}{v_{Pt}\coth(t_{Pt}/(2l_{Pt}))v_{YIG}\coth(t_{YIG}/(2l_{YIG}))} \tag{17}$$

and

$$\frac{1}{v_{eff}} = \frac{1}{v_{Pt}\tanh(t_{Pt}/l_{Pt})} + \frac{1}{v_{YIG}\tanh(t_{YIG}/l_{YIG})} \tag{18}$$

For a bulk YIG with $t_{YIG} \gg l_{YIG}$ the previous equations are simplified as

$$v_p = \frac{v_{Pt}\tanh(t_{Pt}/l_{Pt}) + v_{YIG}}{\tanh(t_{Pt}/(2l_{Pt}))\tanh(t_{Pt}/l_{Pt})} \tag{19}$$

and

$$\frac{v_p}{v_{YIG}} = \frac{(v_{Pt}/v_{YIG})\tanh(t_{Pt}/l_{Pt}) + 1}{\tanh(t_{Pt}/(2l_{Pt}))\tanh(t_{Pt}/l_{Pt})} \tag{20}$$

With $l_{Pt} \simeq 7.3$ nm and $l_{YIG} \sim 0.4\,\mu$m and $v_{YIG} \sim v_{Pt}$. [2]. The value obtained is $v_p/v_{YIG} \simeq 8$ which is close to the measured value of 9.

# Supplementary note 3: SPE and SSE hysteresis loop

The evaluation of the SPE and SSE coefficients is performed with the sample at magnetic saturation, however it is interesting to measure the SPE and SSE at lower magnetic fields, i.e. along the hysteresis loops of YIG as the experimental method employed can work at any applied magnetic field $|H_z| < |H_s|$. In the SPE case, the heat current $I_{q,SP}$ has to be determined by the inversion of the sign of the electric current $\pm I_{e,y}$ in the Pt. The signal offset (see Methods) is determined at $I_{e,y} = 30$ mA with the magnetic field inversion at saturation and is then subtracted from the $I_{q,SP}$ determined by the periodic inversion $I_{e,y} = \pm 30$ mA as a function of a slowly varying magnetic field. The result is shown in Supplementary figure 1 bottom. In the SSE case, the heat current was fixed at 280mW and the voltage offset was determined once by a magnetic field inversion at saturation. The value was then used to correct the voltage measures as a function of the magnetic field. The result is the hysteresis loop of Supplementary figure 1 top. The two loops shows excellent agreement with each other, confirming that we are observing the same phenomenon. It is worth to mention that the rather strange loop shape is the result of the fact that only a small portion of the YIG close to the Pt is active in the spin caloric effects. This thickness can be roughly estimated as the magnetic moment diffusion length within YIG, i.e. 0.4 $\mu$m. The shape of the hysteresis loop at $|H|$ below 10 mT, displays a low field behavior that can be interpreted in terms of the difference between the surface and bulk magnetization YIG as previously observed in Ref.[3].

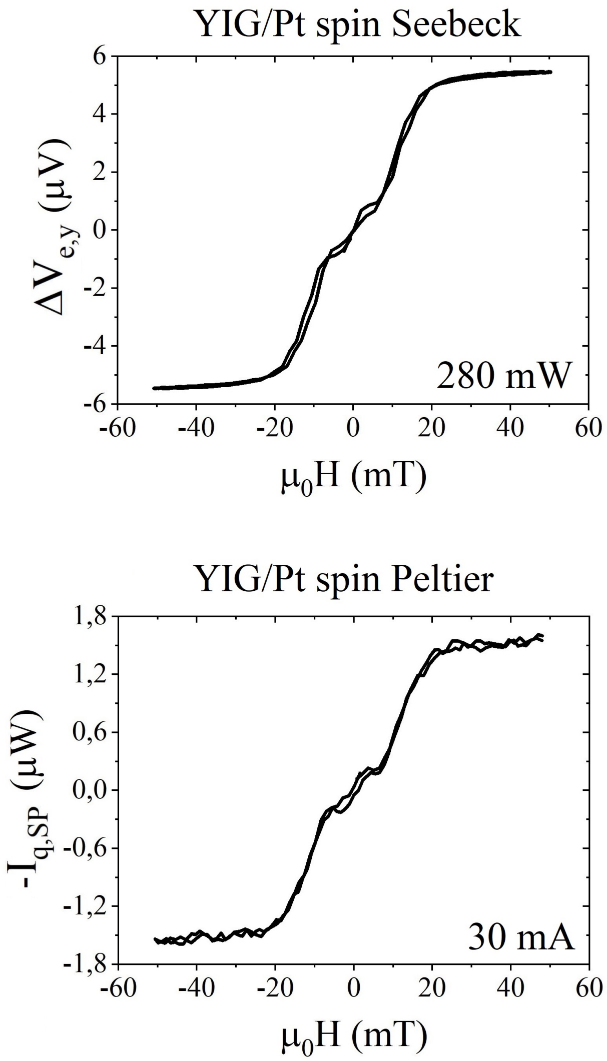


Supplementary figure 1: SSE and SPE hysteresis loops. Top: SSE voltage, $\Delta V_{e,y}$, versus magnetic field $\mu_0 H$ measured at a heat current $I_{q,x} = 280$ mW. Bottom: SPE heat current, $-I_{q,SP}$, versus magnetic field $\mu_0 H$ measured at an electric current of $I_{e,y} = 30$ mA.

# References

[1] V. Basso, E. Ferraro, A. Sola, A. Magni, M. Kuepferling, and M. Pasquale. Nonequilibrium thermodynamics of the spin seebeck and spin peltier effects. *Phys. Rev. B*, 93:184421, 2016.

[2] V. Basso, M. Kuepferling, A. Sola, P. Ansalone, and M. Pasquale. The spin seebeck and spin peltier reciprocal relation. *IEEE Magnetics Letters*, 2018.

[3] K. Uchida, J. Ohe, T. Kikkawa, S. Daimon, D. Hou, Z. Qiu, and E. Saitoh. Intrinsic surface magnetic anisotropy in y3fe5o12 as the origin of low-magnetic-field behavior of the spin seebeck effect. *Physical Review B*, 92:014415, 2015.
